# Supplementary material for: Evidence for absence of links between striatal dopamine synthesis capacity and working memory capacity, spontaneous eye-blink rate, and trait impulsivity
Source: eLife. 2023 Apr 21;12:e83161. doi: 10.7554/eLife.83161 (PMC10162803; doi:10.7554/eLife.83161)
Supplement: Supplementary file 1. [file elife-83161-supp1.docx]

**Supplementary Table 1. Participants characteristics (N=94).**

| Characteristic |  |
| --- | --- |
| Age | range: 18-43  mean (SD): 23.0 (5.0) years |
| Sex | 47 female, 47 male |
| Highest education level | University/higher professional education: 79  Secondary vocational education: 2  Secondary education (high school): 13 |
| Current occupation | Employed: 10  Student: 79  Other: 5 |
